# Wnt Signaling Is Required for Early Development of Zebrafish Swimbladder

**DOI:** 10.1371/journal.pone.0018431

**Published:** 2011-03-30

**Authors:** Ao Yin, Svitlana Korzh, Cecilia L. Winata, Vladimir Korzh, Zhiyuan Gong

**Affiliations:** 1 Department of Biological Sciences, National University of Singapore, Singapore, Singapore; 2 Laboratory of Fish Developmental Biology, Genes and Development Division, Institute of Molecular and Cell Biology, Singapore, Singapore; Laboratoire Arago, France

## Abstract

**Background:**

Wnt signaling plays critical roles in mammalian lung development. However, Wnt signaling in the development of the zebrafish swimbladder, which is considered as a counterpart of mammalian lungs, have not been explored. To investigate the potential conservation of signaling events in early development of the lung and swimbladder, we wish to address the question whether Wnt signaling plays a role in swimbladder development.

**Methodology/Principal Findings:**

For analysis of zebrafish swimbladder development, we first identified, by whole-mount in situ hybridization (WISH), *has2* as a mesenchymal marker, *sox2* as the earliest epithelial marker, as well as *hprt1l* and *elovl1a* as the earliest mesothelial markers. We also demonstrated that genes encoding Wnt signaling members Wnt5b, Fz2, Fz7b, Lef1, Tcf3 were expressed in different layers of swimbladder. Then we utilized the heat-shock inducible transgenic lines hs:Dkk1-GFP and hs:ΔTcf-GFP to temporarily block canonical Wnt signaling. Inhibition of canonical Wnt signaling at various time points disturbed precursor cells specification, organization, anterioposterior patterning, and smooth muscle differentiation in all three tissue layers of swimbladder. These observations were also confirmed by using a chemical inhibitor (IWR-1) of Wnt signaling. In addition, we found that Hedgehog (Hh) signaling was activated by canonical Wnt signaling and imposed a negative feedback on the latter.

**Significance/Conclusion:**

We first provided a new set of gene markers for the three tissue layers of swimbladder in zebrafish and demonstrated the expression of several key genes of Wnt signaling pathway in developing swimbladder. Our functional analysis data indicated that Wnt/β-catenin signaling is required for swimbladder early development and we also provided evidence for the crosstalk between Wnt and Hh signaling in early swimbladder development.

## Introduction

The common evolutionary origin of the teleost swimbladder and tetrapod lung has been long recognized, but the vast anatomical and functional differences between the two organs weaken the common origin assumption [1]. Whereas the swimbladder is a simple gas sac positioned at the dorso-anterior part of the body cavity [2] , the mammalian lung is a much more complicated structure with complex branching morphogenesis [3]. Molecular mechanisms regulating development of the latter have been extensively studied [4]; however, little is known about the molecular events and mechanisms of swimbladder development in fish. Thus, molecular evidence for the evolutionary links between fish swimbladder and tetrapod lung remains to be explored.

Wnt signaling pathway has been reported to play critical roles in mammalian lung development [5]. Early studies have shown that Wnt signaling only plays roles in late lung development by regulating lung epithelium and mesenchyme proliferation. Whereas loss of β-catenin or overexpression of Wnt inhibitor *dkk1* in lung epithelium after lung specification inhibits distal airway epithelial development and a global proximalization [6], mesenchyme-specific inhibition of β-catenin results in reduced mesenchymal proliferation [7], [8]. Lung epithelium-specific loss of *Wnt7b* abrogates distal lung bud formation and perturbs branching morphogenesis [9]. *Wnt7b* is also required for lung smooth muscle differentiation [10] and mesenchymal proliferation [11]. Inactivation of *Wnt5a* acting in the non-canonical Wnt pathway [12] leads to thickening of the mesenchyme and excessive branching of the epithelial airway [13]. *Wnt11* is expressed in the mouse lung, but its function is still not clear [14]. Recently, it has been shown that Wnt signaling is also required for lung endoderm specification and progenitor fate determination [15]. *Wnt2/2b* double knock-out leads to complete lung agenesis in mice due to loss of endodermal progenitor specification, but did not affect other endoderm-derived organs such as thyroid, liver, and pancreas. Furthermore, activation of Wnt/β-catenin signaling leads to the reprogramming of esophagus and stomach endoderm to a lung progenitor fate [15]. Besides Wnt ligands, other Wnt pathway members such as antagonist Dkk1 [9], Frizzled receptors [16] and Lef1/Tcf3 transcription factors [13] also play pivotal roles in mouse lung development.

The mechanisms of zebrafish endoderm specification have been extensively explored [17]. Compared to other endodermal organs, such as the liver [18], [19] and pancreas [20], [21], the swimbladder development has been much less characterized. To date the expression of several genes has been shown in the swimbladder [22], [23], [24], but only Hedgehog signaling and *pbx1* have been linked to development of this organ [25], [26].

Previously, we have identified gene markers for all the three tissue layers of the zebrafish swimbladder, including *hp9* for epithelium, *fgf10a* and *acta2* for mesenchyme and smooth muscle, and *annxa5* for outer mesothelium [26]. In this study, we identified an additional set of gene markers for all three tissue layers, including *sox2* as the earliest epithelial marker, *has2* as a mesenchymal marker, *hprt1l* and *elovl1a* as the earliest outer mesothelial markers. We then showed that components of the Wnt signaling pathway, including *wnt5b*, *fz2*, *fz7b*, *Lef1* and *tcf3*, were expressed in different tissue layers of swimbladder. By using the two heat-shock inducible transgenic zebrafish lines, *Tg(hsp70l:dkk1-GFP)^w32^* (hs:Dkk1-GFP for short in this report) [27] and *Tg(hsp70l:tcf3-GFP)^w26^* (hs:ΔTcf-GFP for short in this report) [28], both of which inhibits the canonical Wnt signaling with the former inhibiting the Wnt signaling by binding of Dkk1 with Lrp5/6 co-receptor [29] and the latter by overexpression of a dominant-negative form of Tcf3 transcription factor. Wnt signaling was blocked at various time points by heat-shock treatments and we observed perturbations to precursor cells specification, organization and patterning in all three tissue layers of the swimbladder.

## Results

### Identification of a new set of gene markers for different tissue layers of zebrafish swimbladder

Our previous study reported the identification of molecular markers such as *hb9*, *fgf10a*, *acta2* and *annxa5* for all the three tissue layers of zebrafish swimbladder [26]. However, the interactions among multiple signaling pathways in developmental context are complex and frequently a particular gene is regulated by more than one pathway or more than one gene. To ensure that the absence of a marker gene expression faithfully reflects a swimbladder defect rather than simply a down-regulation of its expression, it is desirable to use multiple gene markers to trace tissue changes. Thus, we made an effort to identify a new set of gene markers for the three tissue layers of zebrafish swimbladder. Based on the ZFIN online database [30], we first investigated in detail expression pattern of several candidate genes and confirmed the following genes as new markers for swimbladder: *sox2*, *has2*, *hprt1l* and *elovl1a*.

**Figure 1:**
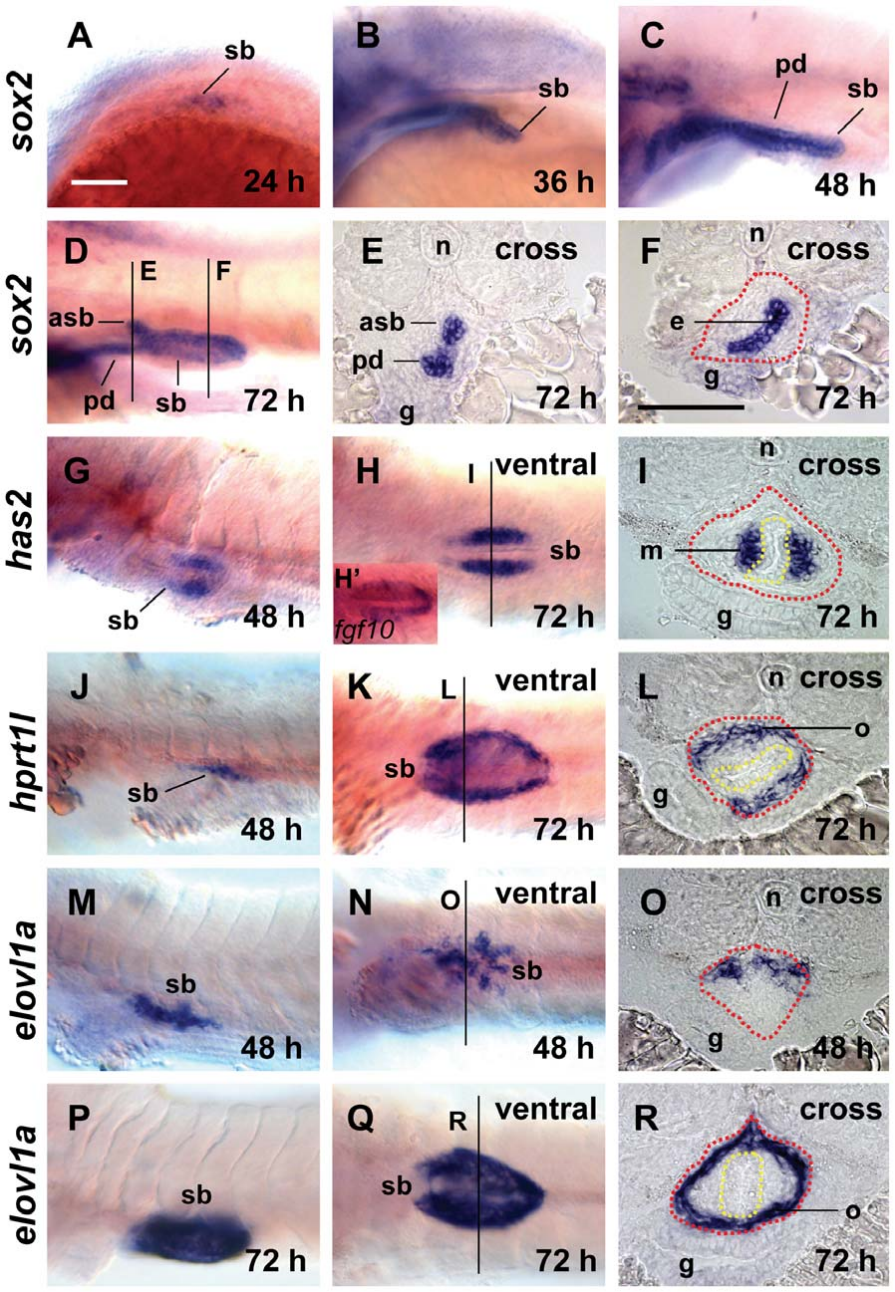
Expression of new maker genes in different tissue layers of the zebrafish swimbladder as assayed by WISH. (A–F) Expression of *sox2* in the epithelium of swimbladder at 24 hpf (A), 36 hpf (B), 48 hpf (C) and 72 hpf (D–F). Panels (A–D) are lateral view while (E,F) are cross sections of the embryo shown in (D) with the section planes indicated. (G–I) Expression of *has2* in the mesenchyme layer of swimbladder at 48 hpf (G, lateral view) and 72 hpf (H, ventral view; I, cross section). (H′) Expression of *fgf10a* in swimbladder (ventral view) for comparison of *has2* expression in (H). (J–L) Expression of *hprt1l* in the outer mesothelium of swimbladder at 48 hpf (J, lateral), and 72 hpf (K, ventral; L, cross section). (M–R) Expression of *elovl1a* in the outer mesothelium of swimbladder at 48 hpf (M, lateral; N, ventral; O, cross section) and 72 hpf (P, lateral; Q, ventral; R, cross section). Dotted red circles indicated swimbladder and yellow circles indicated epithelium. All embryos were laterally oriented with anterior to the left unless specified. Abbreviations: asb, anterior swimbladder bud; e, epithelium; g, gut; m, mesenchyme; n, notochord; o, outer mesothelium; pd, pneumatic duct; sb, swimbladder. Scale bar = 200 µm. Panel (A) scale bar applies to all whole mount images and Panel (F) scale bar is for all cross section images.

Expression of *sox2* was initiated from 24 hpf (Figure 1A) in the endoderm. The expression in swimbladder bud was initiated from 36 hpf, and maintained at 48 hpf and 72 hpf (Figure 1B–D), and restricted to the epithelium (Figure 1E and F). The expression of *sox2* was also present in the pneumatic duct and the anterior swimbladder bud (Figure 1D and E), but absent from any other endoderm organs. Expression of *sox2* from 24 hpf made it the earliest marker for swimbladder epithelium progenitors, which were previously defined at 28 hpf by *pbx1* [25] and *prdc* [31], and at 36 hpf by *hb9* [26].

*has2* expression in the swimbladder was first detected at 48 hpf, and maintained at 72 hpf specifically in the mesenchyme layer (Figure 1G–I). Whereas the previously reported mesenchymal marker *fgf10a* was expressed in the bilateral domain as well as the very posterior domain [26] (Figure 1H′), *has2* expression was only in the bilateral domain (Figure 1H). This difference may indicate that *has2* and *fgf10a* were expressed in different cell lineages in the swimbladder mesenchyme.

*hprt1l* was expressed in the swimbladder from 48 hpf and maintained at 72 hpf specifically in the outer mesothelium (Figure 1J–L). We also found that the swimbladder is the only endodermal organ with *hprt1l* expression. Besides *hprt1l*, we identified another marker, *elovl1a*, for the outer mesothelium and it was expressed at higher level than *hprt1l*. *elovl1a* expression in the swimbladder was detected from 48 hpf in a discrete pattern (Figure 1M and N). Cross section showed that the *elovl1a*-expressing cells were located in the dorsal part of swimbladder primordium (Figure 1O). *elovl1a* expression was maintained at 72 hpf exclusively in the outer mesothelium of swimbladder (Figure 1P–R). Compared to *annxa5*, which expression is initiated only after 60 hpf [26], the expression of *hprt1l* and *elovl1a* starts much earlier (48 hpf) making them the earliest known molecular markers of the outer mesothelium.

### Expression of Wnt pathway members in the swimbladder during early development

**Figure 2:**
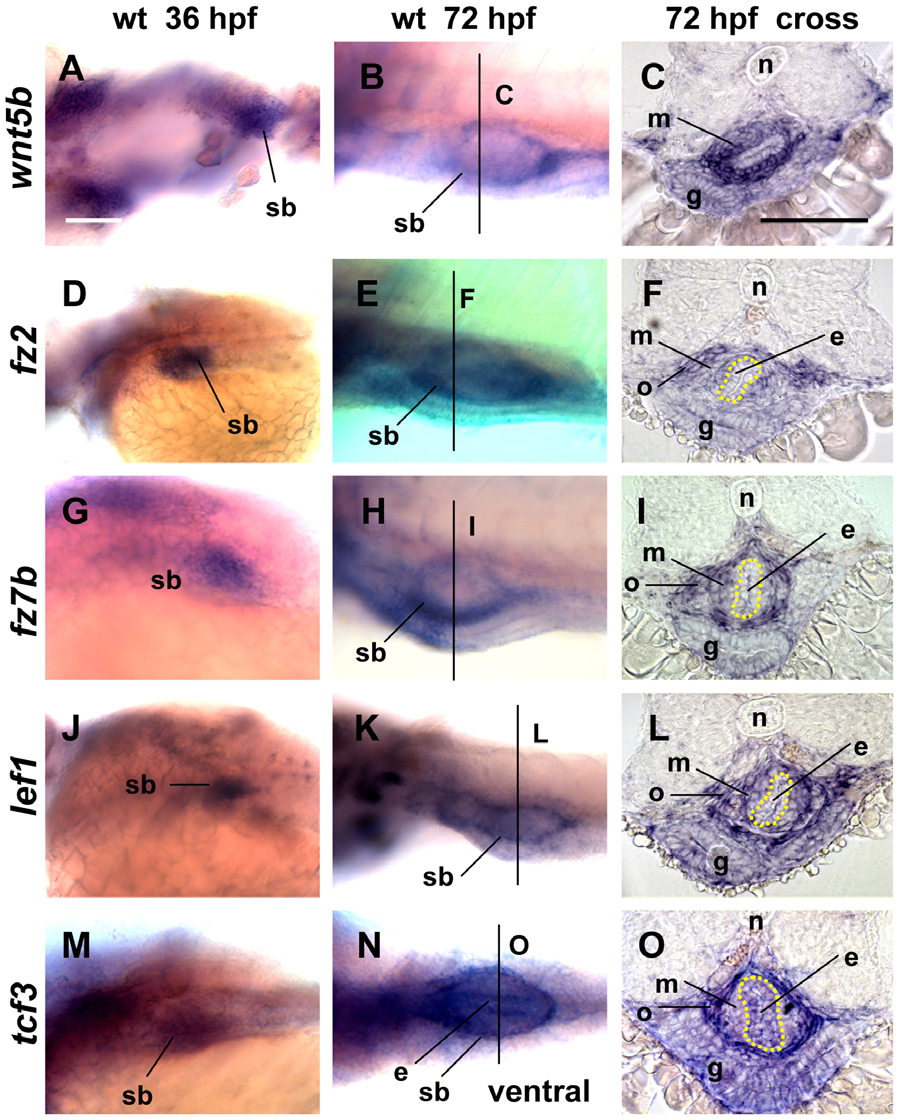
Expression of Wnt pathway genes in zebrafish swimbladder as detected by WISH. (A–C) Expression of *wnt5b* in the mesenchyme of swimbladder at 36 hpf (A) and 72 hpf (B,C). (D–F) Expression of *fz2* in the mesenchyme and outer mesothelium of swimbladder at 36 hpf (D) and 72 hpf (E,F). (G–I) Expression of *fz7b* in mesenchyme and outer mesothelium of swimbladder at 36 hpf (G) and 72 hpf (H,I). (J–L) Expression of *lef1* in the mesenchyme and outer mesothelium of swimbladder at 36 hpf (J) and 72 hpf (K,L). (M–O) Expression of *tcf3* in the epithelium and outer mesothelium of swimbladder at 36 hpf (M) to 72 hpf (N,O). Panels (C, F, I, L, O) are cross sections. All embryos were laterally oriented with anterior to the left unless specified. Dotted red circles indicated swimbladder and yellow circles indicated epithelium. Abbreviations: e, epithelium; g, gut; m, mesenchyme; n, notochord; o, outer mesothelium; pf, pectoral fin; pd, pneumatic duct; sb, swimbladder. Scale bars = 200 µm. Panel (A) scale bar applies to all whole mount images and Panel (C) scale bar is for all cross section images.

To demonstrate that Wnt pathway plays a role in swimbladder development, expression of several genes encoding components of the Wnt pathway was examined. Since *Wnt5a* is expressed in the mouse lung [9] and *Xenopus* lung [32], we examined both *wnt5a* and *wnt5b* expression in zebrafish swimbladder. While *wnt5a* expression was not detected (not shown), *wnt5b* expression was observed in the swimbladder mesenchyme from 36 hpf and maintained at 72 hpf (Figure 2A–C).

In addition to Wnt ligands, we found two receptor genes of Wnt signaling, *fz2* and *fz7b*, were also expressed in the swimbladder (Figure 2). Their expression was detected as early as 36 hpf and maintained at 72 hpf in both mesenchyme and outer mesothelium (Figure 2D–I). The expression of these receptors further supported a role of Wnt signaling in development of swimbladder.

As both a co-activator and a target of Wnt signaling, Lef1 has been used as a reporter for Wnt signaling activity [9]. Our data showed that *lef1* was expressed in the swimbladder from 36 hpf to 72 hpf in the mesenchyme and outer mesothelium (Figure 2J–L), providing another piece of evidence for the activity of Wnt signaling in the swimbladder.

Finally, another co-activator of Wnt target genes, *tcf3* was also expressed in the swimbladder starting from 36 hpf (Figure 2M–O). This expression was strong in the outer mesothelium, moderately weak in the epithelium, and very weak in the mesenchyme layer at 72 hpf (Figure 2O). The presence of *tcf3* expression further supported that Wnt signaling is active during early swimbladder development.

### Inhibition of Wnt signaling by heat-shock of hs:Dkk1-GFP and hs:ΔTcf-GFP transgenic embryos

**Figure 3:**
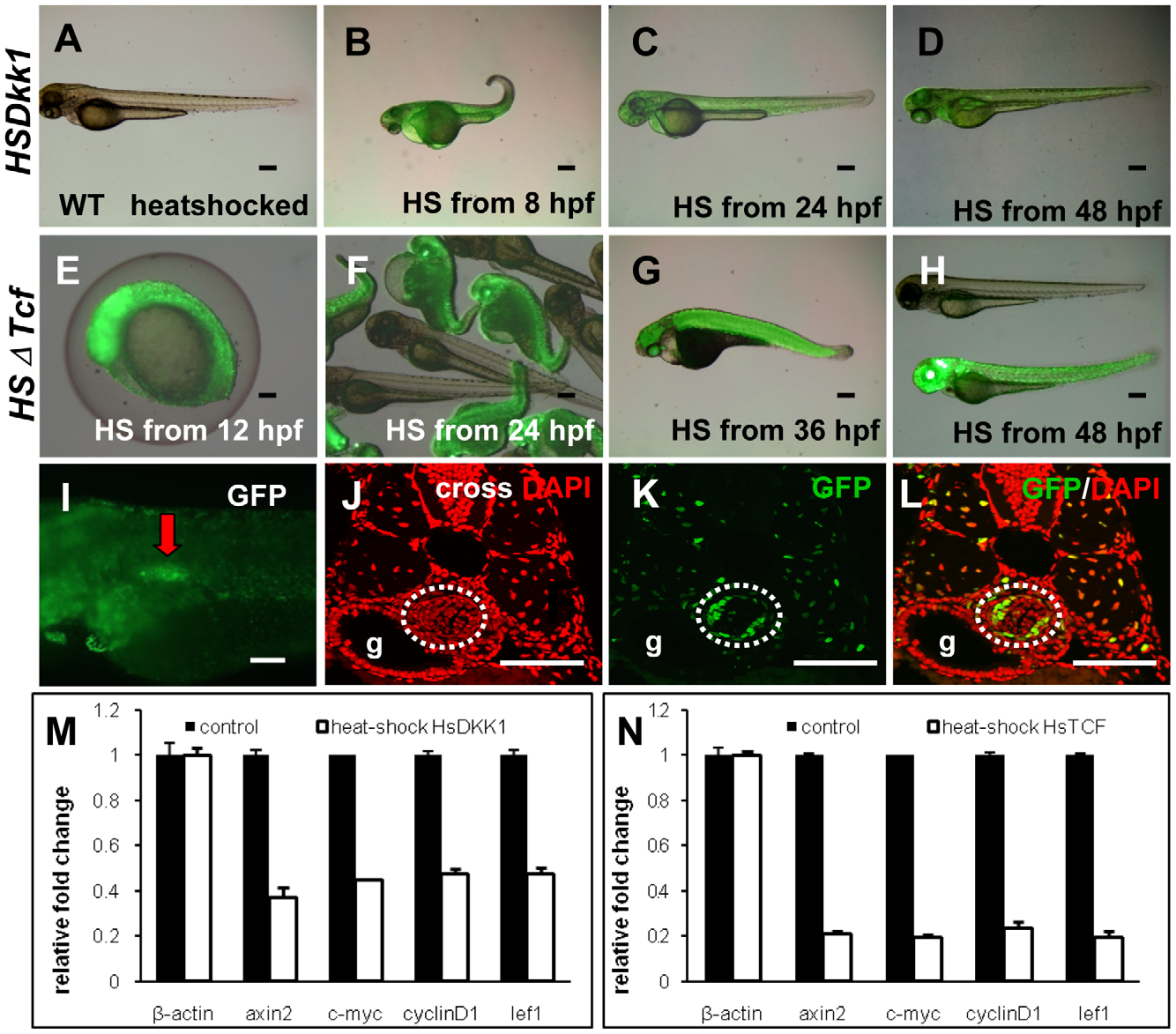
Induction of GFP-fusion proteins and inhibition of Wnt signaling in the hs:Dkk1-GFP and hs:ΔTcf-GFP transgenic embryos by heat-shock treatment. (A–H) Induction of GFP-fusion proteins in hs:Dkk1-GFP and hs:ΔTcf-GFP transgenic embryos. Heat-shock was performed at various stages and live images were taken at 72 hpf. (A) Lack of GFP expression in wild type sibling after heatshock treatment. (B–D) Live image of GFP expression in hs:Dkk1-GFP embryos heat-shocked at 12 hpf (B), 24 hpf (C) and 48 hpf (D). (E–H) Live image of GFP expression in hs:ΔTcf-GFP embryos heat-shocked at 12 hpf (E), 24 hpf (F), 36 hpf (G) and 48 hpf (H). Note that the expression of GFP in hs:ΔTcf-GFP embryos (E–H) were stronger than that of hs:Dkk1-GFP embryos (A–D), implying a stronger inhibition of Wnt signaling in hs:ΔTcf-GFP embryos. (I–L) Analysis of GFP-fusion protein expression in the swimbladder of hs:ΔTcf-GFP transgenic embryos. Transgenic embryos were heat-shocked at 66 hpf and live images were taken at 72 hpf (I), followed by immunohistochemical staining using anti-GFP antibody (K,L) and DAPI counterstaining (J,L). Panels (J–L) are cross sections. (M,N) Real time RT-PCR assays of selected target genes of the Wnt signaling after heat-shock blocking Wnt signaling in hs:Dkk1-GFP (M) and hs:ΔTcf-GFP (N) transgenic embryos. Stronger inhibition of wnt signaling targets genes *axin2*, *c-myc*, *cyclinD1* and *lef1* in hs:ΔTcf-GFP fishes (M) than hs:Dkk1-GFP fishes (N) were observed (p<0.05). All embryos were lateral oriented with anterior to the left unless specified. Dotted white circles indicated swimbladder. Abbreviations: g, gut. Scale bars = 200 µm.

To investigate the functions of Wnt signaling in zebrafish swimbladder development, we utilized two transgenic lines, hs:Dkk1-GFP [27] and hs:ΔTcf-GFP [28], which have been used to block canonical Wnt/β-catenin signaling. Whereas DKK1 acts as a potent inhibitor by binding to Wnt receptors LPR5/6, TCF3 serves as a key transcription factor that regulates numerous Wnt/β-catenin target genes [5]. To ensure that heat-shock treatment of transgenics induced GFP-tagged protein expression, we heat-shocked the embryos at different developmental stages and demonstrated strong GFP expression in all stages from 12 hpf to 48 hpf in both transgenic lines (Figure 3A–H). In particular, GFP-tagged Tcf was strongly induced in the swimbladder (Figure 3I). Immunohistofluorescence (IHF) staining confirmed the induction of GFP expression in all of the three layers of the entire swimbladder (Figure 3J–L). It is interesting to note that more mesenchymal cells than epithelial and outer mesothelial cells were induced to express GFP (Figure 3J–L). Similar GFP expression patterns were observed in hs:Dkk1-GFP fishes (not shown). Our data from quantitative real-time PCR using previously reported target genes *axin2*, *c-myc*, *cyclinD1* and *lef1* [5] revealed that heat-shock led to a 50% and 80% loss of Wnt activity in hs:Dkk1-GFP and hs:ΔTcf-GFP fishes respectively (Figure 3M and N).

### Stage-specific inhibition of Wnt signaling impaired the swimbladder development in the epithelium

**Figure 4:**
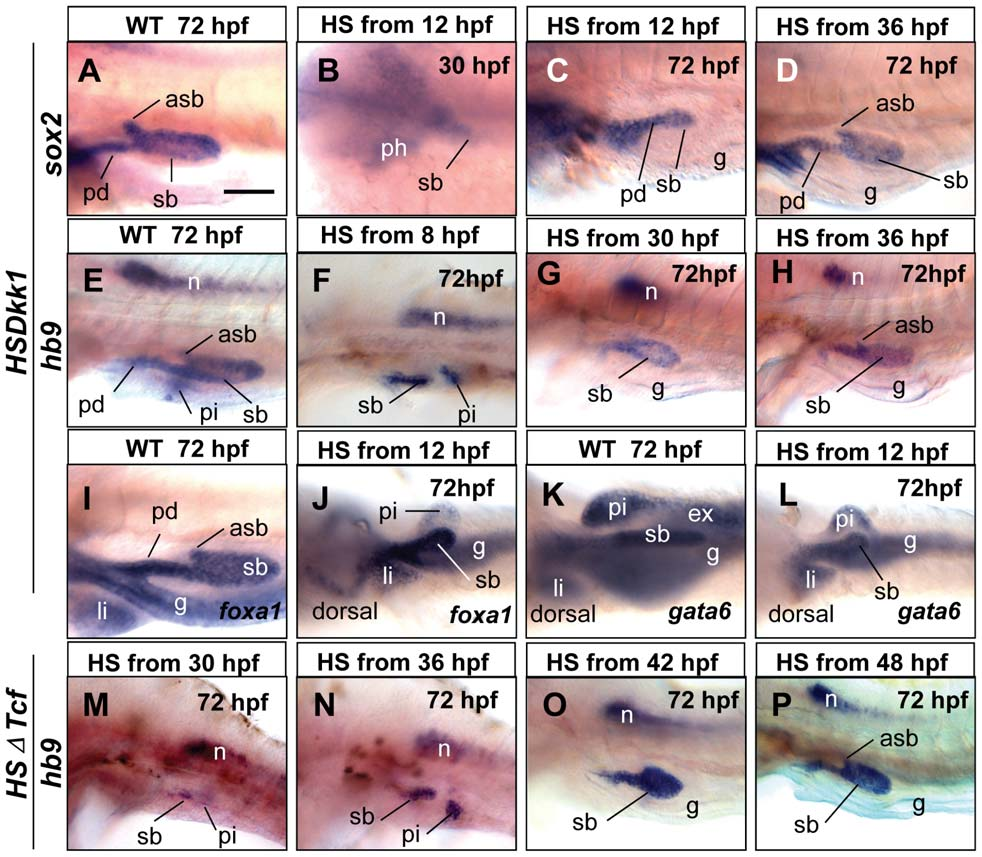
Effects of temporal inhibition of wnt signaling on the epithelium development of swimbladder. (A–L) Expression of marker genes in hs:Dkk1-GFP embryos after heat-shock treatment at various stages. (A–D) Expression of *sox2* in swimbladder epithelium in wild type sibling at 72 hpf (A) and in hs:Dkk1-GFP embryos heat-shocked from different developmental stages as indicated (B–D). Expression of *sox2* in the swimbladder epithelium anlage at 30 hpf when heat-shocked from 12 hpf (B). (E–H) Expression of *hb9* at 72 hpf in wild type swimbladder epithelium, in 72 hpf hs:Dkk1-GFP larvae heat-shocked from 8 hpf, 30 hpf (G) and 36 hpf (H). (I, J) Expression of *foxA1* at 72 hpf in the epithelium of endoderm organs in wild type and transgenic larvae heat-shocked from 12 hpf (J). (K, L) Expression of *gata6* in the epithelium of endoderm organs at 72 hpf in wild type and transgenic fishes heat-shocked from 12 hpf. (M–P) Expression of *hb9* in the epithelium of swimbladder at 72 hpf in transgenic larvae heat-shocked from 30 hpf, 36 hpf, 42 hpf and 48 hpf. Note the presence of anterior swimbladder bud (asb) in (D, H, P). All embryos were laterally oriented with anterior to the left unless specified. Abbreviations: asb, anterior swimbladder bud; ex, exocrine pancreas; g, gut; li, liver; n, notochord; pd, pneumatic duct; ph, pharynx; pi, pancreatic islet; sb, swimbladder. Scale bar in (A) = 200 µm for all panels.

The effect of inhibition of Wnt signaling on the development on the swimbladder epithelium was first examined through heat-shock of the hs:Dkk1-GFP and hs:ΔTcf-GFP embryos at different developmental stages and development of the epithelium was monitored by using *sox2* and *hb9* as markers. Heat-shock of hs:Dkk1-GFP embryos from as early as 8 hpf (Figure 3B), a time point in the late gastrulation, did not abrogate epithelial specification. The epithelial precursors of swimbladder and pancreatic islet were specified, although their number was severely reduced (Figure 4F). Heat-shock of hs:Dkk1-GFP embryos from 12 hpf led to 6-hour delay in the specification of epithelial precursors at 30 hpf (Figure 4B), and the formation of a reduced epithelial bud at 72 hpf (Figure 4C). Whereas heat-shock of hs:Dkk1-GFP embryos from 30 hpf resulted in reduction of epithelium without the anterior bud at 72 hpf (Figure 4G), heat-shock from 36 hpf resulted in a well formed, though smaller epithelium, including the anterior chamber bud at 72 hpf (Figure 4D and 4H), comparable to the swimbladder phenotype in wild type siblings (Figure 4A and 4E).

Blocking of Wnt signaling using another transgenic line, hs:ΔTcf-GFP, resulted in more severe defects in swimbladder epithelium. Heat-shock from 12 hpf caused all embryos to die before 30 hpf (not shown). The number of epithelial precursors of swimbladder severely decreased at 72 hpf when heat-shock was performed from 30 hpf (Figure 4M), but the swimbladder was mildly affected and formed a morphologically recognizable primordium at 72 hpf when heat-shock was performed from 36 hpf (Figure 4N). When hs:ΔTcf-GFP embryos were heat-shocked from 42 hpf, the swimbladder was well-formed but without the anterior bud (Figure 4O). In contrast, a complete (but much reduced) swimbladder epithelium including the anterior swimbladder bud was formed at 72 hpf when larvae were heat-shocked from 48 hpf (Figure 4P).

To examine the effects of Wnt blocking on other endodermal organs, we investigated the heat-shocked larvae by WISH using *foxa1* and *gata6* markers expressed in all endodermal tissues. At 72 hpf, hs:Dkk1-GFP larvae that were heat-shocked from 12 hpf showed a smaller swimbladder bud and liver, normal pancreatic islet, and absence of exocrine pancreas (Figure 4I–L).

### Blocking of Wnt signaling perturbed mesenchyme development and smooth muscle differentiation

**Figure 5:**
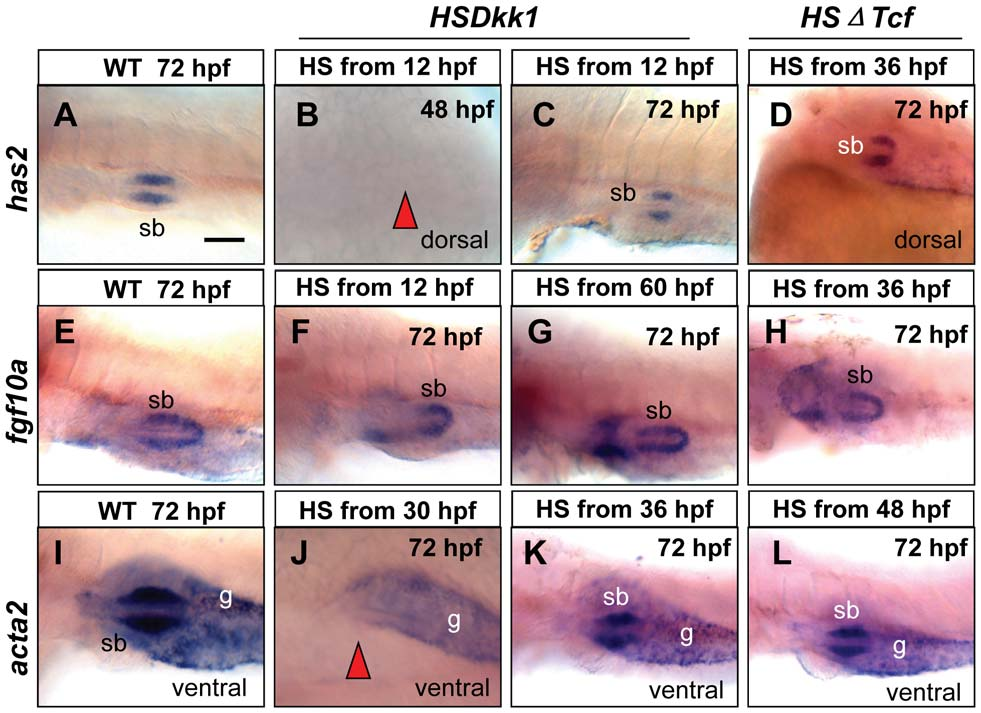
Effects of temporal inhibition of Wnt signaling on swimbladder mesenchyme and smooth muscles. (A, C) Expression of *has2* in swimbladder mesenchyme at 72 hpf in wild type and hs:Dkk1-GFP fishes that were heat-shocked from 12 hpf. (B) Absence of *has2* expression at 48 hpf in the putative swimbladder when hs:Dkk1-GFP larvae were heat-shocked from 12 hpf. (D) Expression of *has2* in the size-reduced mesenchyme in hs:ΔTcf-GFP larvae heat-shocked from 36 hpf. (E–G) Expression of *fgf10a* at 72 hpf in mesenchyme of wild type (E), transgenic hs:Dkk1-GFP larvae heat-shocked from 12 hpf (F) and 60 hpf (G). (H) Expression of *fgf10a* in the size-reduced mesenchyme in hs:ΔTcf-GFP larvae heat-shocked from 36 hpf. (I–K) Expression of *acta2* at 72 hpf in mesenchyme of wild type, hs:Dkk1-GFP larvae heat-shocked from 30 hpf and 36 hpf. Note absence of *acta2* staining in (J) and the reduced size of swimbladder in (K). (L) Expression of *acta2* in the size-reduced mesenchyme in hs:ΔTcf-GFP larvae heat-shocked from 48 hpf. Scale bar in (A) = 200 µm for all panels.

Effects of Wnt signaling inhibition on mesenchyme development were also observed. When the hs:Dkk1-GFP embryos were heat-shocked from 12 hpf, the mesenchyme was still absent at 48 hpf (Figure 5B) but appeared at 54 hpf (not shown), indicating a 6-hour delay of mesenchyme specification. By 72 hpf, the mesenchyme was well-formed, but much smaller in size (Figure 5A, C and E, F). Heat-shock at 60 hpf resulted in an almost normal mesenchyme (Figure 5G). In the hs:ΔTcf-GFP transgenics, swimbladder mesenchyme was absent at 72 hpf when the transgenic embryos were heat-shocked from 30 hpf (not shown); however, the mesenchyme was present when the transgenic embryos embryos were heat-shocked from 36 hpf (Figure 5D and H). These observations indicated that Wnt signaling is required for the specification of swimbladder mesenchyme in a specific developmental window.

To examine the effects of Wnt signaling on differentiation of smooth muscle, we used WISH to detect the smooth muscle marker *acta2*. In the hs:Dkk1-GFP fishes, smooth muscle differentiation was totally abolished when fishes were heat-shocked from 30 hpf or earlier (Figure 5J). Smooth muscles were present but the size of mesenchyme was reduced when larvae were heat-shocked from 36 hpf (Figure 5I and K). A similar phenotype was observed in hs:ΔTcf-GFP larvae heat-shocked from 48 hpf (Figure 5L). Therefore, Wnt signaling, similar to Hh signaling [26], is required for mesenchyme cells differentiating into smooth muscles.

### Blocking of Wnt signaling disturbed the outer mesothelium development

**Figure 6:**
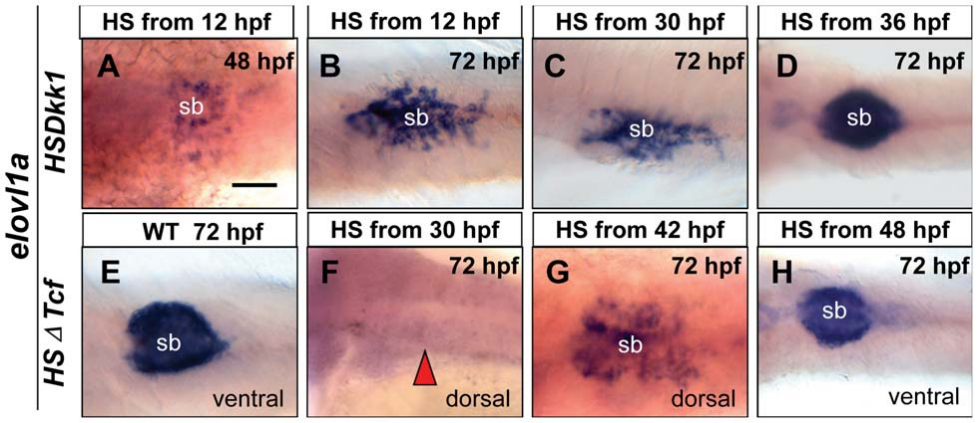
Effects of temperal inhibition of Wnt signaling on swimbladder mesothelium development. (A, B) Expression of *elovl1a* in outer mesothelium at 48 hpf and 72 hpf when hs:Dkk1-GFP fishes were heat-shocked from 12 hpf. The *elovl1a* expressing cells were disorganized at 72 hpf when larvae were heat-shocked from 24 hpf (C), but were well organized with smaller size when larvae were heat-shocked from 30 hpf (D). (E) Expression of *elovl1a* in wild-type outer mesothelium. The *elovl1a* expressing cells in the outer mesothelium at 72 hpf were absent when hs:ΔTcf-GFP larvae were heat-shocked from 30 hpf (F), were present but disorganized when larvae were heat-shocked from 42 hpf (G), and were properly organized but with smaller size when fishes were heat-shocked from 42 hpf (H). All embryos were laterally oriented with anterior to the left unless specified. Red arrows indicated absence of swimbladder at putative locations. Abbreviations: g, gut; sb, swimbladder. Scale bar in (A) = 200 µm for all panels.

To investigate the impact of Wnt signaling on development of outer mesothelium, we performed WISH with *elovl1a* as a marker of this tissue layer in heat-shocked hs:Dkk1-GFP and hs:ΔTcf-GFP transgenics. In hs:Dkk1-GFP fishes, mesothelial cells were present at 48 hpf when heat-shocked from 12 hpf (Figure 6A). When transgenics were heat-shocked at 12 hpf and 30 hpf, the cluster of mesothelial cells was abnormally extended along the A–P axis at 72 hpf (Figure 6B and C).

Heat-shock from 36 hpf resulted in an organized but smaller mesothelium at 72 hpf (Figure 6D and E). Heat-shock of hs:ΔTcf-GFP transgenics from 30 hpf, the mesothelial cells were absent at 72 hpf (Figure 6F). Heat-shock from 36 and 42 hpf led to the disorganized cells (Figure 6G) and later treatment from 48 hpf resulted in a well organized albeit size-reduced mesothelium (Figure 6H). Therefore, these data suggested that Wnt signaling was required for both specification and organization of outer mesothelium cells of swimbladder.

### Knockdown of Wnt signaling led to reduced cell proliferation and enhanced apoptosis in swimbladder

**Figure 7:**
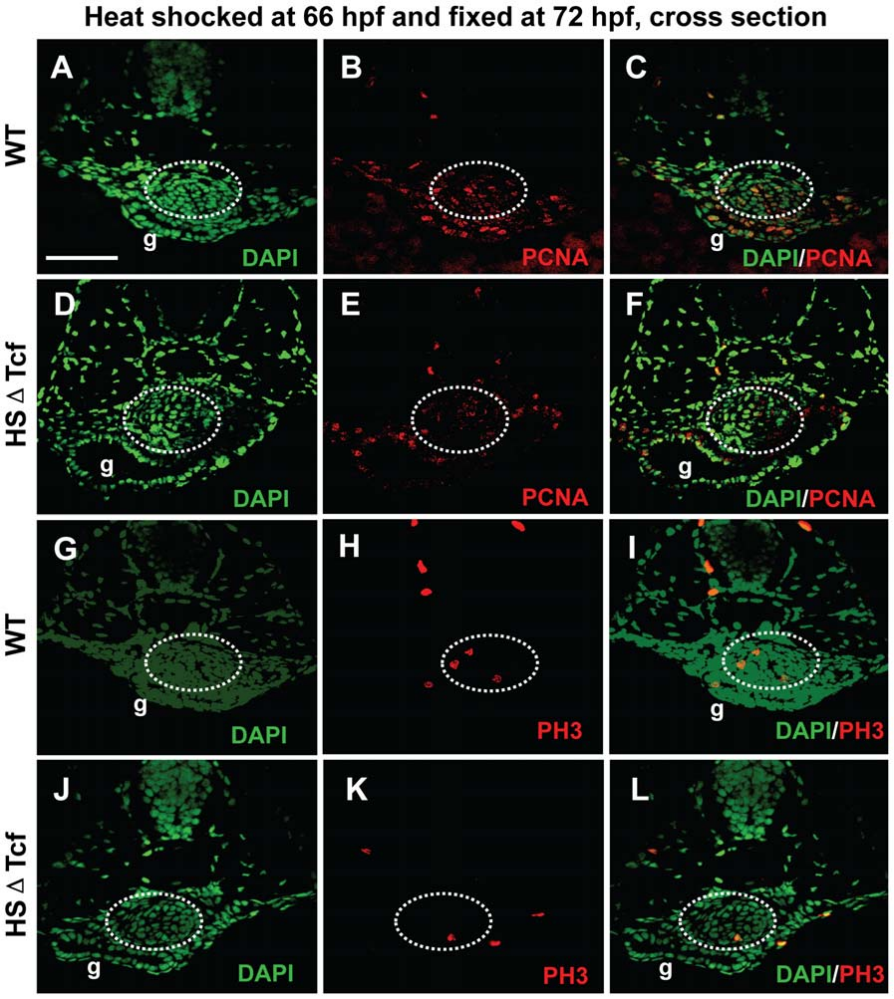
Effects of Wnt inhibition on cell proliferation in swimbladder. hs:ΔTcf-GFP fish was out-crossed with AB wild type fish, the resultant heterozygous embryos and their wild type siblings were heat-shocked at 66 hpf and fixed at 72 hpf for proliferation assay. (A–F) Proliferation assay detecting PCNA-positive cells (red) with DAPI counterstaining (green). The number of PCNA-positive cells (red) was greatly reduced in transgenic fishes (D–F) (n = 5) compared to that of controls (A–C) (n = 5). (G–L) Staining for phosphorylated histone H3 (PH3, red) with DAPI counterstaining (green). Compared to wild type fishes (G–I) (n = 5), the number of PH3-positive cells (red) was greatly reduced in transgenic fishes (D–F) (n = 5). Dotted white circles indicated swimbladder. Abbreviation: g, gut. Scale bar = 200 µm for all panels.

**Figure 8:**
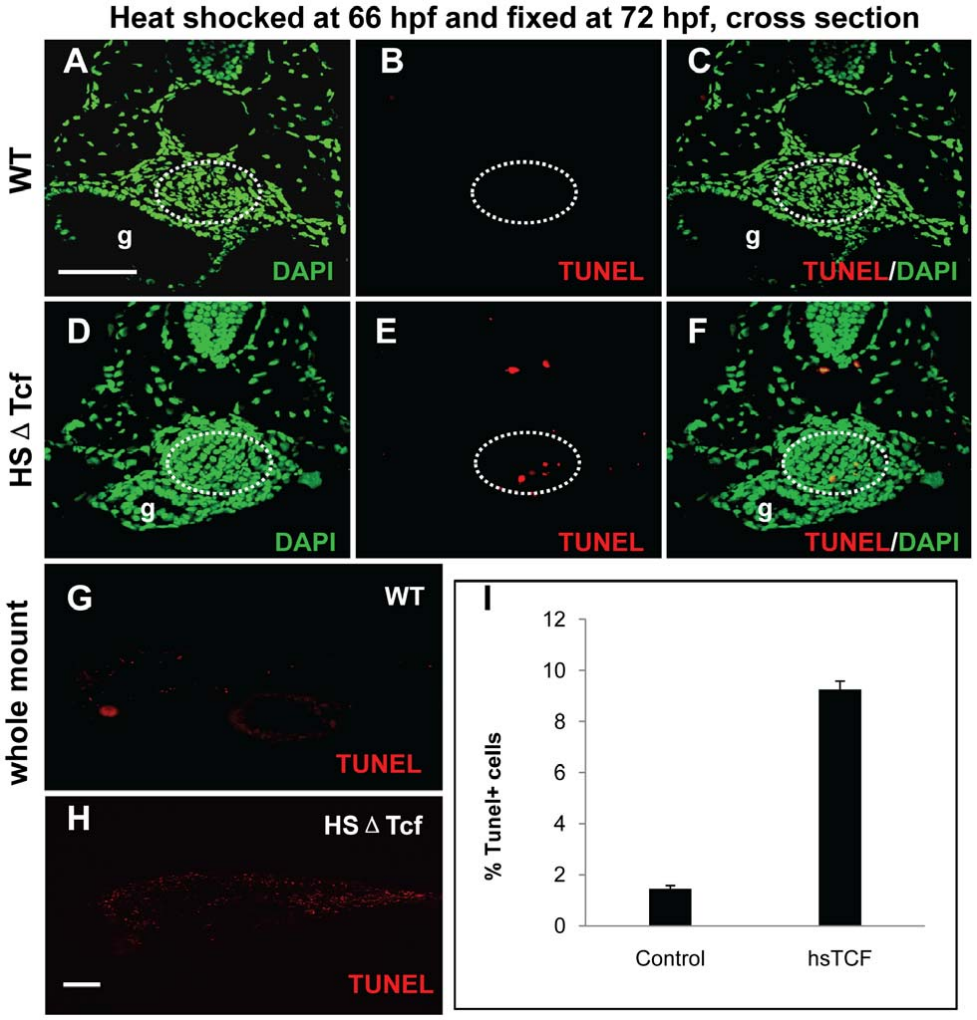
Effects of Wnt inhibition on cell apoptosis in swimbladder. hs:ΔTcf-GFP heterozygous larvae and their wild type siblings were heat-shocked at 66 hpf and fixed at 72 hpf for TUNEL assay. (A–F) TUNEL-positive cells (red) and DAPI-stained cells (green) shown in cross section. The number of TUNEL-positive cells in swimbladder greatly increased in transgenic fishes (D–E) (n = 11 of 13, 85%) compared to that of controls (A–C) (n = 9 of 10, 90%). (G, H) Imaging of whole mount TUNEL-stained (red) transgenic fishes (H) and controls (G). (I) The statistics assays showed that the percentage of apoptosis cells in heat-shocked transgenics increased from 1.45% to 9.26% (I) (p = 0.01). Note the globally increased number of TUNEL-positive cells in (H). All embryos were laterally oriented with anterior to the left. Dotted white circles indicated swimbladder. Abbreviation: g, gut. Scale bar = 200 µm for all panels.

To explore the mechanisms that affect swimbladder development by blocking of Wnt signaling, we examined the effects of Wnt inhibition on cell proliferation and apoptosis. In the meantime, to ensure the alteration of cell proliferation and apoptosis are direct effects of Wnt blocking, we adopted the protocol used in zebrafish study previously [27]. Wild type and hs:ΔTcf-GFP embryos were heat-shocked at 66 hpf and analyzed immediately at 72 hpf. The PCNA-positive cells in the swimbladder of transgenic embryos were reduced (Figure 7A–F). Similar results were also observed when the anti-phosphorylated histone H3 (PH3) antibody was used to detect cells in M-phase of cell cycle (Figure 7G–L). Therefore, Wnt signaling is required for cell proliferation in zebrafish swimbladder. In contrast, the TUNEL-positive cells increased globally (Figure 8G and H). In the swimbladder region, apoptotic cells were dramatically increased in heat-shocked transgenics (Figure 8A–F). Our statistical data showed that the percentage TUNEL-positive cells against total cells in heat-shocked transgenic fish swimbladder increased by nearly eight folds compared to that of heat-shocked wild type embryos (Figure 8I). Therefore, blocking of Wnt signaling dramatically promoted cell apoptosis in swimbladder. Taken together, in agreement with previous reports based on *in vitro* studies [33], the defective swimbladder development caused by blockade of Wnt signaling was partially due to reduction of cell proliferation and increase of apoptosis.

### Crosstalk between Wnt and Hh signaling in swimbladder development

**Figure 9:**
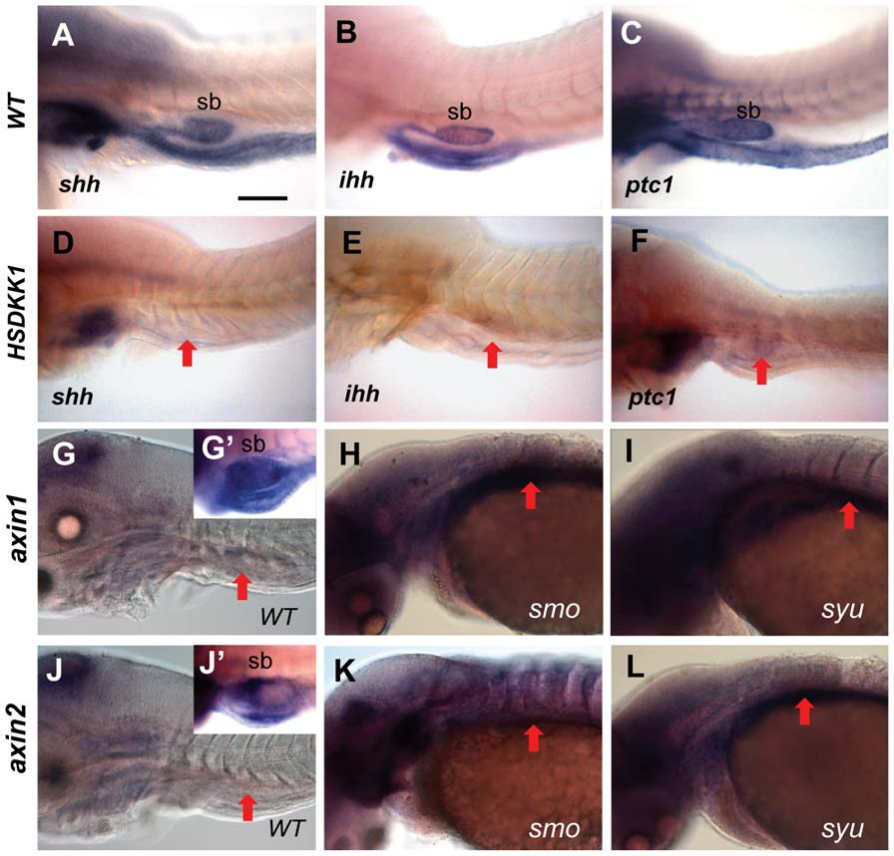
Crosstalk of Wnt and Hh signalling in swimbladder development. (A–C) Expression of *shh* (A), *ihh* (B) and *ptc1* (C) in swimbladder of controls at 72 hpf. These controls were heat-shocked at 66 hpf and fixed at 72 hpf prior to WISH. (D–F) Decreased expression of *shh* (D), *ihh* (E) and *ptc1* (F) in swimbladder of hs:Dkk1-GFP fry after heat-shock. (G–I) Expression of *axin1* in wild type (G), *smo^b641^* (H) and *syu^t4^* (I) at 72 hpf. (J–L) Expression of *axin2* in wild type (J), *smo^b641^* (K) and *syu^t4^* (L) at 72 hpf. Embryos in (G–L) were short-stained for the same one hour for comparison and no detectable *axin1* and *axin2* signals were observed in controls but prolonged staining (5 hours) showed enriched hybridization signals in the swimbladder in controls (G′ and J′). Compared to the short-staining pictures in (G) and (J), increased global expression of *axin1* and *axin2* in *smo^b641^* and *syu^t4^* was observed although the swimbladder development has been severely affected in the two mutants [26]. Red arrows indicate the position of swimbladder. Abbreviation: sb, swimbladder. Scale bar in (A) = 200 µm for all panels.

Since crosstalk between Wnt and Hh signalings have been reported in regulation of mammalian lung development [11], [34], it is interesting to investigate whether these pathways interact in the regulation of swimbladder development in zebrafish. The expression levels of genes encoding Hh signaling components including *shh*, *ihh* and *ptc1* [26] in the swimbladder were greatly reduced in heat-shocked hs:Dkk1-GFP embryos (Figure 9D–F) compared to those in wild type control (Figure 9A–C). These results suggested that Wnt signaling is required to maintain the expression of Hh components in the swimbladder during these early stages.

We then investigated whether Hh signaling regulates Wnt signaling in swimbladder development by examination of the expression of Wnt target genes, *axin1*, *axin2* and *lef1* in two Hh pathway mutants, *smo^b641^*, in which Hh signaling is completely deprived due to a mutation in the co-receptor gene *smoothened* [35], and *syu^t4^*, which is partially deficient in Hh signaling due to *shh* mutation [36]. We demonstrated that *axin1* and *axin2* were expressed in the swimbladder from 36 hpf (not shown) to 72 hpf (Figure 9G′ and J′, long staining for 5 hours). Then we found that the global expression of *axin1* and *axin2* were substantially enhanced at 72 hpf in both *smo^b641^* (Figure 9H and K) and *syu^t4^* (Figure 9I and L) mutants compared to that in the wild type controls (Figure 9G and J) for the same one hour short staining. The elevation of *axin1* and *axin2* expression was mainly observed in the central nervous system and somites as lack of Hh signaling greatly hinders the development of swimbladder [26]. Thus the observed increase of Wnt signaling in the absence of Hh signaling indicates the requirement of Hh signaling for maintaining appropriate levels of Wnt signaling. Taken together, these data implied that Hh signaling is maintained by Wnt signaling and plays a negative feedback loop on Wnt signaling during the early swimbladder development. Whereas Wnt5a represses *shh* expression and Wnt7a signaling has no interaction with Shh signaling in mouse lung development [11], [34], there is no report on the effect of Shh signaling on Wnt signaling. Our current data from analyses of seimbladder development may provide some new clues for the interaction between Wnt and Hh signaling in lung development.

### Confirmation of Wnt signaling blocking by a chemical inhibitor and a model of Wnt signaling requirement in swimbladder morphogenesis

**Figure 10:**
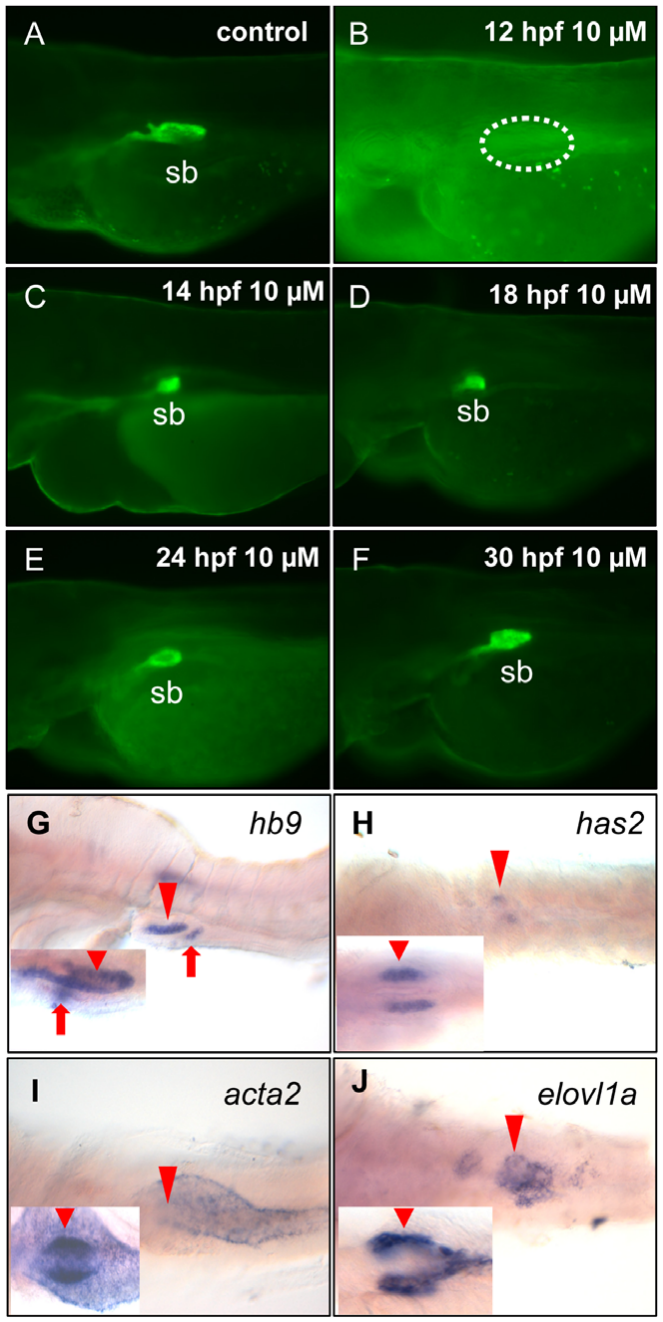
Chemical inhibition of Wnt signaling and its effect on swimbladder development. (A–F) Effect of Wnt inhibition on development of swimbladder epithelia. *Et(krt4:EGFP)^sq33-2^* embryos were doped with 10 µM IWR-1 from various time points and then live-imaged at 72 hpf. Panel (A) shows normal swimbladder epithelium in a controls without IWE-1 treatment and panels (B–F) show the lack of swimbladder epithelium in embryos treated from 12 hpf and smaller swimbladders in embryos treated from 14 hfp (C), 16 hpf (D), 24 hpf (E) and 30 hpf (F). Dotted circle indicates the position of swimbladder. (G–J) Effect of Wnt signaling inhibition on development of different tissue layers of swimbladder. Control embryos were treated with 10 µM IWR-1 from 14 hpf and were assayed by WISH at 72 hpf. The swimbladder epithelium, mesenchyme, smooth muscle and mesothelium were marked by *hb9* (G) , *has2* (H), *acta2* (I) and *elvol1a* (J) expression respectively. Inserted boxes present their expression in normally developed swimbladder in controls for comparison. (G) shows lateral view and (H–J) show ventral view. The red arrowheads indicate the swimbladder, whereas the red arrows indicate pancreas. Abbreviation: sb, swimbladder.

To further demonstrate the requirement of Wnt signaling in early swimbladder development, the small molecule IWR-1, a potent and specific antagonist of Wnt signling by targeting the components that function downstream of Lrp and Dvl proteins, was employed to suppress Wnt signaling and its potency has been recently tested in fin regeneration in adult zebrafish [37]. To examine its effect on zebrafish swimbladder development, *Et(krt4:EGFP)^sq33-2^* embryos, which displayed GFP fluorescence in the swimbladder epithelia [26], were used to incubate with different concentrations of IWR-1 starting from 12 hpf, a time point critical for the specification or cell fate decision of the swimbladder epithelial precursor cells. As shown in Fig. S1, these embryos displayed a dosage-dependant effect in swimbladder specification. At 1 µM and 5 µM IWR-1, the specification of swimbladder was not affected (Fig. S1A–C). When the concentration was increased to 10 µM and 20 µM, the specification of the swimbladder epithelia was completely abrogated (Fig. S1D and E). Thus, by using the chemical inhibitor, the requirement of the Wnt signaling for the three tissue layers of swimbladder morphogenesis was examined (Fig. 10). When *Et(krt4:EGFP)^sq33-2^* embryos were treated with 10 µM IWR-1 from 12 hpf, the swimbladder epithelium was missing as observed at 72 hpf (Fig. 10B), compared to untreated *Et(krt4:EGFP)^sq33-2^* embryos at 72 hpf (Fig. 10A). However, when embryos were treated with 10 µM IWR-1 from 14 hpf (Fig. 10C), 18 hpf (Fig. 10D), 24 hpf (Fig. 10E) and 30 hpf (Fig. 10F), a small epithelium bud was observed at 72 hpf. Therefore, the Wnt signaling is critical for the specification of the swimbladder epithelium between 12 hpf and 14 hpf, a time point about two hours earlier than that of Hedgehog requirement [26], implying that Wnt signaling may act upstream of Hh signaling in controlling the specification of the swimbladder epithelium.

WISH assays with molecular markers for different tissue layers were also carried out on the 72-hpf embryos treated with IWR-1 (Fig. 10G–J). When IWR-1 treatments were performed with 10 µM IWR-1 from 14 hpf, specification of of all three tissue layers were observed as evident by *hb9* expression in epithelia (Fig. 10G), *has2* expression in mesenchyme (Fig. 10H) and *elov1a* expression in mesothelium (Fig. 10J), but they all became smaller and disorganized. The smooth muscle differentiation was completely abrogated (*acta2* expression in Fig. 10I). These observations are similar to earlier observations by using the heat-shock inducible transgenic lines to block Wnt signalings (Figs. 4, 5, 6).

**Figure 11:**
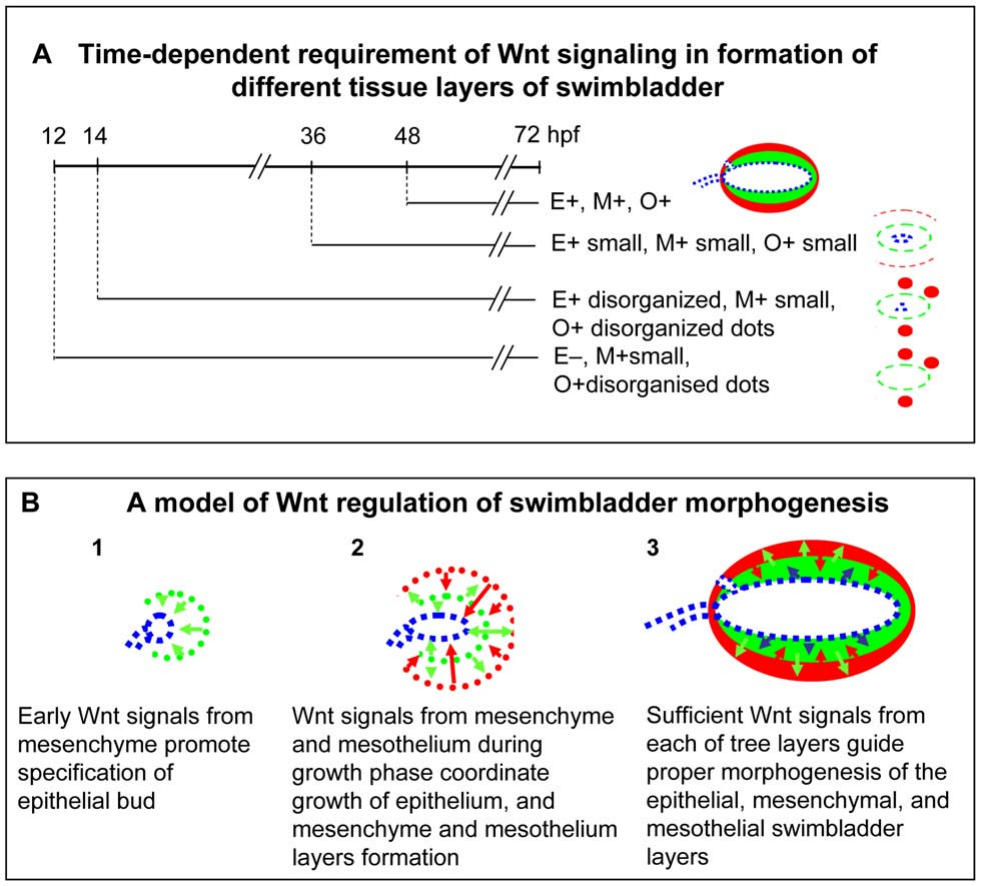
Schematic representation of Wnt signaling requirement in swimbladder development. (A) Time-dependent requirement of Wnt signaling in formation of different tissue layers of swimbladder. Treatments with 10 µM IWR-1 were initiated from different developmental stages and morphologies were summarized on the right. E, M and O indicate epithelium, mesenchyme and outer mesothelium respectively. “+” indicates presence and “−” indicates absence. (B) A proposed model Wnt regulation of swimbladder morphogenesis. (1) Early Wnt signals (green arrows) induce specification of epithelial cells. (2) Wnt signals secreted by mesenchyme (green arrows) and mesothelium (red arrows) coordinate the organization and growth of three layers. (3) Sufficient Wnt signaling secreted from epithelium (blue arrows), mesenchyme (green arrows) and mesothelium (red arrows) guide proper growth of the three tissue layers.

Based on the information from both the heat-shock transgenic lines and chemical inhibitor IWR-1, the time-dependent requirement of Wnt signaling is summarized in Fig. 11A. When Wnt signaling was blocked from 12 hpf, the epithelium specification was abrogated, whereas the mesenchyme and mesothelium were specified, with the latter disorganized. When Wnt signaling was inhibited from 14 hpf, the epithelium was specified but disorganized, and the mesothelium and mesenchyme remained disorganized. Blocking of Wnt signaling from later time points at 36 hpf and 48 hpf resulted in properly organized but smaller swimbladder rudiments in all three tissue layers.

Based on these observations, a model of regulation of Wnt signaling on swimbladder morphogenesis is proposed (Fig. 11B). Early Wnt signaling from mesenchyme precursors determines the specification of epithelium. Wnt signaling from mesenchyme and mesothelium during growth stage coordinates the organization of epithelium and mesothelium, as well as growth of all three layers. Wnt signals at subsequent growth stage from all three layers regulate proper morphogenesis of all the three tissue layers of swimbladder.

## Discussion

In the current study, we first identified a new set of molecular markers for all the three tissue layers of zebrafish swimbladder, with *sox2* as the earliest epithelial marker, *hprt1l* and *elovl1a* as the earliest mesothelium markers, and *has2* as a new mesenchyme marker. We then demonstrated that a number of genes encoding Wnt signaling members, including *wnt5b*, *fz2*, *fz7b*, *lef1*, *tcf3* were expressed in different layers of swimbladder. By using the two heat-shock inducible Wnt inhibition transgenic zebrafish lines, hs:Dkk1-GFP and hs:ΔTcf-GFP, we demonstrated that the expression of GFP-tagged Dkk1/Tcf was induced in the swimbladder. Subsequent conditional blocking of Wnt signaling at various developmental stages showed that the precursor cells specification, organization, and patterning in were all perturbed in the three tissue layers. Furthermore, we demonstrated that the decreased cell proliferation and increased apoptosis contributes to the disturbance of swimbladder growth. In addition, our data suggest that Hh signaling was maintained by Wnt signaling in the swimbladder but it also acted in a feedback loop to inhibit Wnt signaling. Therefore, our study for the first time established the critical roles of Wnt/β-catenin signaling in zebrafish early swimbladder development.

### Wnt signaling is required for formation of the bud of anterior chamber of swimbladder

The bud of anterior chamber of swimbladder is formed at 60 hpf and inflated at around 20 dpf (day post-fertilization) when a fully functional swimbladder is developed [26]. Although the mechanisms including Hh [26] and Wnt (this study) signalings that regulate development of the main swimbladder chamber were investigated, no effort has been exerted to investigate the mechanisms involved in formation of the anterior swimbladder bud. In this study, we found that the anterior swimbladder bud was affected by deficiency in Wnt signaling. In hs:Dkk1-GFP embryos heat-shocked from 30 hpf or earlier, the anterior bud was missing (Figure 4C and 4G), but was formed when they were heat-shocked from 36 hpf or later (Figure 4D and 4H). Similarly in hs:ΔTcf-GFP embryos, the anterior chamber bud was absent when transgenic embryos were heat-shocked from 42 hpf or earlier (Figure 4O) and was properly formed when heat-shock was initiated from 48 hpf or later (Figure 4P). Taken together, these results suggested that Wnt signaling is essential for the specification and morphogenesis of the bud of anterior chamber of swimbladder. This finding is reminiscent of the roles of Wnt signaling in mouse lung branching morphogenesis, where inhibition of canonical Wnt signaling led to decreased branching [9], [13]. Thus, the budding of anterior chamber in fish swimbladder could be a primitive event of branching morphogenesis and our study further reinforced the conserved role of Wnt signaling in branching morphogenesis between lung and swimbladder.

### Crosstalk among different tissue layers during early swimbladder development

It has been shown that growth of the mouse lung epithelium and mesenchyme is coordinately regulated by Wnt7b [11]. The crosstalk between the epithelium and mesenchyme of zebrafish swimbladder has also been revealed by the functions of Hh signaling [26]. To investigate if Wnt signaling also plays a role in the crosstalk, we analyzed the timing of manifestation of deficiency in each tissue layer of the swimbladder. Firstly, we examined the relationship between the epithelium and mesenchyme. When the epithelium was severely reduced (Figure 4M), the mesenchyme cells were missing (not shown). When the epithelium bud was formed (Figure 4N), the mesenchyme cells were present and properly organized (Figure 5D and 5H). These observations suggested that specification and organization of mesenchyme required a critical number of the epithelial cells, which is in concert with our previous report on a role of Hh signaling in development of swimbladder [26]. Secondly, we examined the relationship between epithelium and differentiation of smooth muscle. Whenever the epithelium was not fully organized (Figure 4G and 4O), the mesenchyme cells failed to differentiate into smooth muscle (Figure 5J). Only when the full structure of epithelium was formed (Figure 4D and 4P), smooth muscle differentiation occurred (Figure 5K and 5L). These results therefore implied that fully organized epithelium is required for smooth muscle differentiation in the mesenchyme, in accordance with previous report [26]. Thirdly, we examined the relationship of epithelium and mesenchyme in organization of the outer mesothelium. The mesothelial cells did not appear (Figure 6F) until the mesenchyme cells were specified (Figure 5D and 5H). This implies that in this case the specification of mesothelial cells is dependent on the specification of mesenchymal cells, in comparison to the Hh signaling, which is required not only for specification but also for organization and proliferation of mesothelial cells [26]. The correlation of the disorganization of outer mesothelium (Figure 6C and 6G) with the incomplete epithelium (Figure 4G and 4O) and the proper organization of mesothelium (Figure 6D and 6H) with the well-formed epithelium (Figure 4D, 4H and 4P) suggests that the proper patterning of the outer mesothelium by the canonical Wnt signaling depends much more on the proper organization of the epithelium than that in case of Hh signaling [26]. Finally, in agreement with our previous report [26], the concurrent occurrence of the proper organization of the outer mesothelium (Figure 6C–D and 6G–H) and the differentiation of smooth muscles (Figure 5J–L) may imply that its organization requires the differentiation of smooth muscles. Taken together, in addition to addressing the crosstalk between epithelium and mesenchyme [11], [26], our study revealed the crosstalks between epithelium/mesechyme and the outer mesothelium.

## Materials and Methods

### Ethics statement

All experimental protocols were approved by Institutional Animal Care and Use Committee (IACUC) of National University of Singapore (Protocol 079/07) and Institute of Molecular and Cell Biology of Singapore (IMCB).

### Zebrafish strains and heat-shock treatment

Wild type zebrafish were from AB background. The heterozygous hs:Dkk1-GFP [27] and hs:ΔTcf-GFP [28] transgenic lines were obtained from Dr. Randall T. Moon through Dr. Sudipto Roy. Embryos were grown in egg water with 0.2 mM 1-phenyl-2-thiourea (PTU) to prevent pigmentation. The embryos were heat shocked from different time points, i.e., 8, 12, 18, 24, 30, 36, 42, 48, 54 and 66 hpf; the heat-shock was performed in 38°C water bath for 1 hour and repeated twice a day. Wild-type siblings from out-crosses served as controls and were mixed with transgenics to undergo the same heat-shock treatment. Each heat-shock treatment and subsequent assays were performed in three independent experiments.

### Whole mount in situ hybridization (WISH)

WISH was performed using digoxigenin (DIG)-labeled antisense RNA probes as described previously [38]. Embryos at desired stages were fixed with 4% paraformamide (PFA) overnight at room temperature (RT). After washing off PFA with 4×15 min washes in PBST (Phosphate Buffered Saline - Tween 20), the fishes were treated with Proteinase K (PK). The 24, 36, 48 hpf embryos were treated In 1 µl PK/ml PBST for 15 min, 25 min and 45 min respectively. The 60 and 72 hpf embryos were treated in 2 µl PK/ml PBST for 45 min and 50 min respectively. The embryos were then refixed with 4% PFA for 30 min at room temperature, followed by 4×15 min washes in PBST. The embryos were pre-hybridized in hybridization buffer at 67°C for 6 hour or overnight, followed by indefinite storage at −20°C until use. For probe synthesis, total RNA was isolated from 40 AB fish embryos at various stages (6, 24, 36, 48, 72 hpf) using the QIAGEN RNeasy Mini Kit. 0.5 µg of resultant total RNA was used as template for RT-PCR using QIAGEN Onestep RT-PCR Kit. The following PCR primers were used to amplify templates for specific probes: *has2* (1120 bp amplicon) F: CCTGGAGGACTGGTATGATC; R: CACACAATGCTAACACAACCAC; *hprt1l*: (870 bp) F: GAAGCAGCACAGAATCAGGC; R: CTCGTTCGCACCAAGTGTG; *elovl1a* (1070 bp) F: CTTGCTGGGATACGTCTTCTC; R: GATGCTGTCAGGTGTCAGAG. Amplified fragments were ligated into pGEM-T Easy Vector (Promega), following by sequencing confirmation based on gene sequences in GeneBank: *has2* (NM_153650); *hprt1l* (NM_001002056); *elovl1a* (NM_001005989). Sequence-verified clones were used to synthesize DIG-labeled probes using SP6 or T7 RNA polymerase (Ambion) for 3 hours at 37°C. 2 µl of DNase I (Roche) was then added to each reaction, incubated at 37°C for 15 min to remove template plasmid. The reactions were then purified with QIAGEN RNeasy Mini Kit. Other probes including are *sox2*, *wnt5b*, *fz2*, *fz7b*, *lef1* and *tcf3* from lab stock. The probes were diluted in hybridization buffer to a concentration of 1 ng/µl. 1 ml of diluted probes was pre-absorbed with 20 pre-hybridized embryos at 68°C overnight. The pre-absorbed probes were then stored at −20°C until use. DIG-labeled riboprobes in embryos were detected with alkaline phosphatase (AP)-conjugated anti-DIG antibody (Roche) followed by staining with NBT/BCIP (Nitro-Blue Tetrazolium Chloride/5-Bromo-4-Chloro-3′-Indolyphosphate p-Toluidine Salt) to produce purple precipitate. Stained embryos were post-fixed in 4% PFA for 1 hour and washed 3×10 min in PBST. Finally the embryos were in indefinitely kept in 50% glycerol in PBS at 4°C for clarification until imaging.

### Cell proliferation and apoptosis assay

For phosphorylated histone H3 (PH3) assay, zebrafish larvae were fixed in 4% PFA in Phosphate-buffered saline (PBS) overnight at 4°C. For PCNA proliferation analysis, larvae were fixed with Histochoice (Amresco H120, USA) for 1 hour at RT. The embryos were cryo-sectioned at a thickness of 10 µm. Slides were incubated in mouse anti-PCNA (1∶100; Dako M0879, Denmark) and rabbit anti-phosphorylated histone H3 (PH3; 1∶200, Millipore 06-570, USA) antibodies overnight at 4°C. Slides were then washed 4×20 min with PBS and then incubated with secondary antibodies (goat-anti-mouse Alexa-fluor 594 for PCNA and goat-anti-rabbit Alexa-fluor 594 for PH3, Invitrogen, USA) for 1–2 hours in dark at RT. Slides were rinsed 4×15 min in dark in PBS. For apoptosis assay, larvae were fixed with 4% PFA overnight 4°C, the 10 µm section slides were incubated in the labeling solution for 1 hour at 37°C in dark, according to the protocol of the In Situ Cell Death Detection Kit TMR Red (Roche, 12156792910, Mannhelm, Germany) and washed 4×20 min in PBS at RT in dark. The slides were mounted with Vectashield DAPI (4′-6-Diamidino-2 phenylindole) mounting media (Vector H1200, USA) to counter-stain the nucleus, nail-polish sealed and kept in dark at RT for immediate microscopy.

### Reverse transcription quantitative real-time PCR (RT-qPCR)

Total RNAs were isolated using RNeasy kit (QIAGEN) and 1 µg of RNA was used for reverse-transcription into first-strand complimentary DNA (fscDNA) using a SuperScript III Reverse Tanscriptase kit (Invitrogen). 2 µl of resultant fscDNA was used for RT-qPCR, following the MIQE (Minimum Information for Publication of Quantitative Real-Time PCR Experiments) guidelines [39]. Briefly, the RT-qPCR was carried out on an Applied Biosystem 7500 Fast machine (Applied Biosystem), using the 2^−ΔΔCt^ SYBR green protocol [40]. Three pairs of primers for each of *axin2*, *c-myc*, *cyclinD1* and *lef1* were designed to produce a cross-intron amplicon around 150 bp and evaluated by regular PCR to choose a good pair that does not form dimmers. One pair of primers for each gene was selected as following: *axin2* (F: 5′ggacacttcaaggaacaactac; R: 5′ cctcatacattggcagaactg3′), *c-myc* (F: 5′taacagctccagcagcagtg3′; R: 5′gcttcaaaactaggggactg3′), *cyclinD1* (F: 5′gccaaactgcctatacatcag3′; R: 5′tgtcggtgcttttcaggtac3′), *lef1* (F: 5′gagggaaaagatccaggaac3′; R: 5′aggttgagaagtctagcagg3′). β-actin [41] was used as a reference. The thermo cycles are as following: 50°C 2 min, 95°C 10 min, followed by 40 cycles of 95°C 15 sec and 60°C 1 min. RT-qPCR data were analyzed using the ABI7500Fast software, which validates primer quality by analyzing melting curves. All the RT-qPCRs were repeated three times with triplicates for each treatment.

### Treatment of zebrafish embryos with the chemical inhibitor IWR-1

The small molecule inhibitor of Wnt response (IWR-1) [37] was purchased from Sigma-Aldrich (cat. number: I0161). The 25 mg powder was dissolved in 3.06 ml Dimethyl sulfoxide (DMSO) to prepare a 20 mM stock solution which was stored at −20°C in dark. All the embryos subjected to IWR-1 treatment were dechorionated for full access of chemicals. To treat the embryos at a concentration of 10 µM, 30 embryos were cultured in a 60×15 mm plastic falcon dish containing 10 ml egg water, which was added with 5 µl 20 mM stock solution drop-wise to the area without embryos while swirling the dish until a full dispense of chemicals. The egg water and chemicals were changed once a day until assays. A dish adding with 5 µl DMSO was used as a control to eliminate the possible toxicity conferred by DMSO solvent. All the treatments were performed in triplicates.

### Microscopy

Photography of live embryos and WISH embryos was conducted using a dissecting fluorescent microscope (SZX12 Olympus, Japan) and a compound microscope (Zeiss Axioscope 2, Zeiss, Germany). Microscopy of the sections after immunohistochemical staining was performed using a confocal microscope (Olympus FV1000 Fluoview, Japan).

## Supporting Information

Figure S1 **Dosage-dependent effect of IWR-1 on specification of the swimbladder epithelial cells.** The *Et(krt4:EGFP)^sq33-2^* embryos were cultured in egg water with IWR-1 addition from 12 hpf at a concentration as indicated, and was live-imaged for GFP fluorescence at 72 hpf. (A) Fully developed epithelium of the swimbladder at 72 hpf in a control embryo. (B, C) The small bud of the swimbladder epithelium in embryos treated with 1 µM (B) and 5 µM (C) IWR-1. (D, E) The absence of the swimbladder epithelium at 72 hpf in embryos treated with 10 µM (D) and 20 µM (E) IWR-1. Dotted circles indicate the position of swimbladder. Abbreviations: sb, swimbladder.
(TIF)
