# Supplementary figures and images for: Wnt Signaling Is Required for Early Development of Zebrafish Swimbladder
Source: PLoS One. 2011 Mar 30;6(3):e18431. doi: 10.1371/journal.pone.0018431 (PMC3068184; doi:10.1371/journal.pone.0018431)

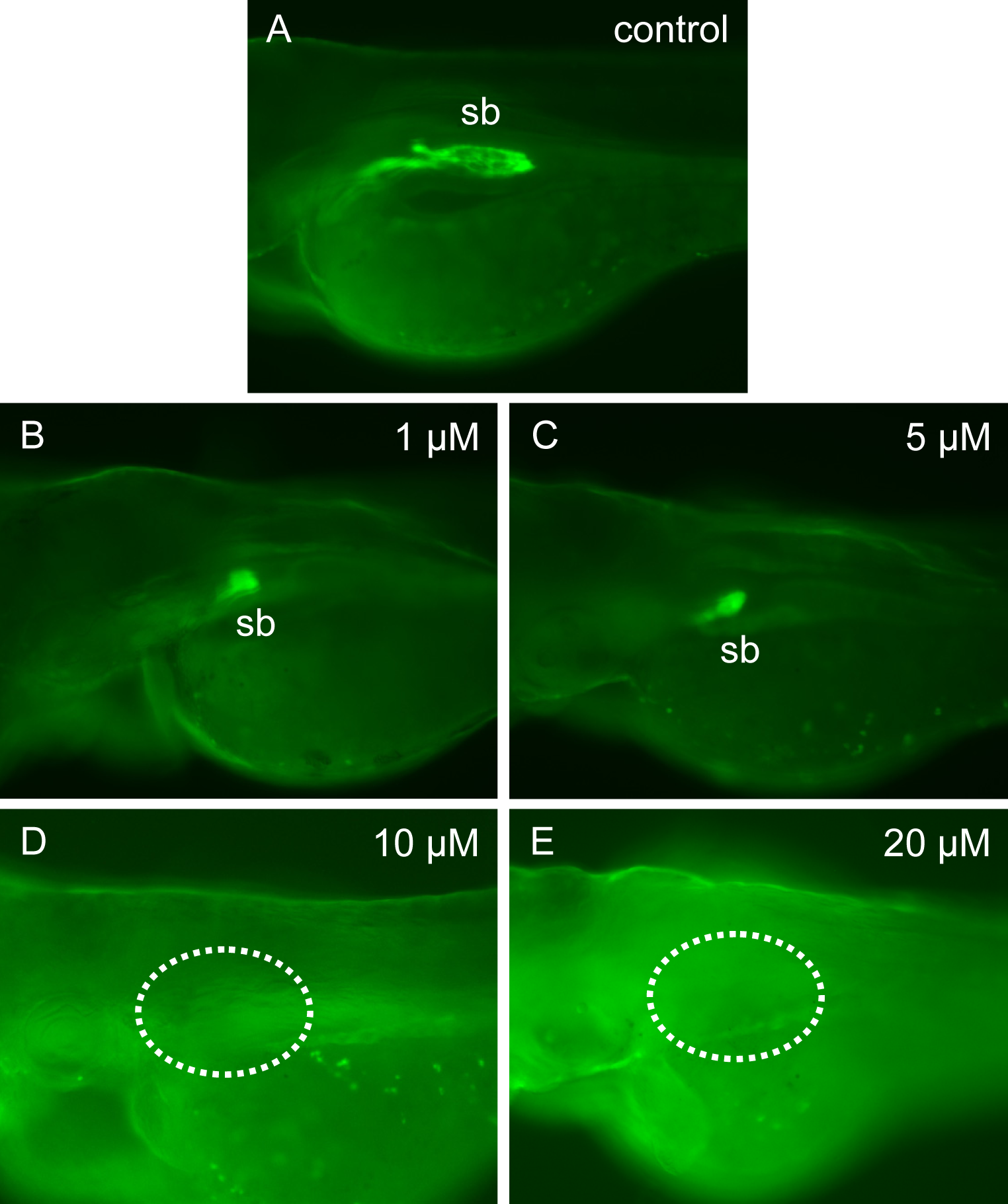

Supplement: Figure S1 — Dosage-dependent effect of IWR-1 on specification of the swimbladder epithelial cells. The Et(krt4:EGFP)sq33-2 embryos were cultured in egg water with IWR-1 addition from 12 hpf at a concentration as indicated, and was live-imaged for GFP fluorescence at 72 hpf. (A) Fully developed epithelium of the swimbladder at 72 hpf in a control embryo. (B, C) The small bud of the swimbladder epithelium in embryos treated with 1 µM (B) and 5 µM (C) IWR-1. (D, E) The absence of the swimbladder epithelium at 72 hpf in embryos treated with 10 µM (D) and 20 µM (E) IWR-1. Dotted circles indicate the position of swimbladder. Abbreviations: sb, swimbladder. (TIF) [file pone.0018431.s001.tif]
